# Development of Ahmedabad’s Air Information and Response (AIR) Plan to Protect Public Health

**DOI:** 10.3390/ijerph15071460

**Published:** 2018-07-10

**Authors:** Vijay S. Limaye, Kim Knowlton, Sayantan Sarkar, Partha Sarthi Ganguly, Shyam Pingle, Priya Dutta, Sathish L. M., Abhiyant Tiwari, Bhavin Solanki, Chirag Shah, Gopal Raval, Khyati Kakkad, Gufran Beig, Neha Parkhi, Anjali Jaiswal, Dileep Mavalankar

**Affiliations:** 1 Natural Resources Defense Council (NRDC), New York, NY 10011, USA; kknowlton@nrdc.org (K.K.); ssarkar@nrdc.org (S.S.); ajaiswal@nrdc.org (A.J.); 2 Mailman School of Public Health, Columbia University, New York, NY 10032, USA; 3 Indian Institute of Public Health, Gandhinagar (IIPH-G), Gandhinagar 382042, India; psganguly@iiphg.org (P.S.G.); shyampingle@iiphg.org (S.P.); priyadutta@iiphg.org (P.D.); sathishlm@iiphg.org (S.L.M.); abhiyant.tiwari@gmail.com (A.T.); dmavalankar@iiphg.org (D.M.); 4 Harvard T.H. Chan School of Public Health, Cambridge, MA 02115, USA; 5 Health Department, Ahmedabad Municipal Corporation (AMC), Ahmedabad 380001, India; bhavinsolanki@ahmedabadcity.gov.in (B.S.); amc.epidemic@gmail.com (C.S.); 6 Apollo Hospital, Ahmedabad 382428, India; 7 Ashrai Associates and Sparsh Chest Diseases Center, Ahmedabad 380009, India; raval_g@yahoo.com; 8 L.G. Hospital, AMC MET Medical College, Ahmedabad 380008, India; kakkad2008@gmail.com; 9 Indian Institute of Tropical Meteorology (IITM), Pune 411008, India; beig@tropmet.res.in (G.B.); neha@tropmet.res.in (N.P.)

**Keywords:** air pollution, exposure mitigation, climate change, India, vulnerability, urban public health, risk communication, community engagement, environmental education, environmental forecasting

## Abstract

Indian cities struggle with some of the highest ambient air pollution levels in the world. While national efforts are building momentum towards concerted action to reduce air pollution, individual cities are taking action on this challenge to protect communities from the many health problems caused by this harmful environmental exposure. In 2017, the city of Ahmedabad launched a regional air pollution monitoring and risk communication project, the Air Information and Response (AIR) Plan. The centerpiece of the plan is an air quality index developed by the Indian Institute of Tropical Meteorology’s System for Air Quality and Weather Forecasting and Research program that summarizes information from 10 new continuous air pollution monitoring stations in the region, each reporting data that can help people avoid harmful exposures and inform policy strategies to achieve cleaner air. This paper focuses on the motivation, development, and implementation of Ahmedabad’s AIR Plan. The project is discussed in terms of its collaborative roots, public health purpose in addressing the grave threat of air pollution (particularly to vulnerable groups), technical aspects in deploying air monitoring technology, and broader goals for the dissemination of an air quality index linked to specific health messages and suggested actions to reduce harmful exposures. The city of Ahmedabad is among the first cities in India where city leaders, state government, and civil society are proactively working together to address the country’s air pollution challenge with a focus on public health. The lessons learned from the development of the AIR Plan serve as a template for other cities aiming to address the heavy burden of air pollution on public health. Effective working relationships are vital since they form the foundation for long-term success and useful knowledge sharing beyond a single city.

## 1. Introduction

### 1.1. Air Pollution as A Public Health Concern in India

Recent comprehensive analyses estimate that globally, exposure to ambient air pollution causes 4.1 million premature deaths each year, largely due to the impacts of small particles on the progression of cardiovascular disease [1]. Of this total, two-thirds of the burden falls in Asia, where ambient concentrations of fine particulate matter (PM_2.5_, particles of aerodynamic diameter ≤ 2.5 microns) are highest [2,3,4,5,6,7]. In India, exposure to PM_2.5_ causes more than half a million premature deaths each year [2,7], and a study of 2016 data found that 14 Indian cities ranked in the top 20 globally for the worst pollution levels [8]. Robust epidemiological research over the past two decades confirms that both acute and chronic exposure to ambient air pollution, especially PM_2.5_, causes many adverse health effects [9,10,11,12,13]. For example, chronic exposure to PM_2.5_ is associated with increased risk of premature mortality, stroke, heart disease, lung cancer, asthma, and other respiratory diseases [6,11,14,15,16,17,18].

Cities are increasingly working to disseminate information on air pollution levels and associated health risks with the aim of reducing harmful exposures at the population level [19,20,21]. One key tool to communicate such data is the air quality index (AQI), which summarizes air quality conditions in a single metric and distills information on associated health risks in a way that is accessible to the public [22,23]. Recent research indicates that AQI systems, when accompanied by emissions reductions on the most polluted days, could help cities achieve cleaner air and tangible health benefits [24]. In 2015, a steering committee organized by the Ministry of Health and Family Welfare issued a comprehensive report that examined India’s air pollution problem from a public health perspective. The report recommended the deployment of both AQI systems and targeted health messaging at the local level as a strategy for addressing the significant health risks posed by polluted air [25]. At the state level, the Gujarat Pollution Control Board (GPCB) is active in addressing air pollution and is developing a long-term clean air plan [26] in parallel with the Ministry of Environment, Forests, and Climate Change (MOEFCC) National Clean Air Programme on air pollution monitoring and control [27]. Complementing these efforts, the city of Ahmedabad is among the first cities in India where city leaders, state government, and civil society are proactively working together to address the air pollution challenge through an exposure mitigation plan centered upon an AQI [28,29,30].

### 1.2. Economic Growth and Climate Change

Continuing economic growth in India and associated demand for energy need not exacerbate the air pollution problem; indeed, cleaner air achieved through the deployment of both renewable energy sources (e.g., wind and solar power) and control technologies (e.g., flue gas desulfurization at thermal coal-fired power plants) improves worker productivity, health, and overall quality of life [31,32,33,34,35,36]. Air quality management requires appropriate control technologies and multi-pronged strategies deployed in targeted sectors. For example, the expansion of India’s middle class is expected to increase the share of two-wheelers and private automobiles on already congested and highly polluted roadways [37,38,39,40,41]. Progressively strengthened vehicle fleet emission standards [42], in tandem with expanding public transportation systems [43,44] and active transportation options [45], can help reduce pollution emissions from the transportation sector [46].

Climate change is expected to directly and indirectly exacerbate already high concentrations of air pollution across India. In addition to PM_2.5_, ground-level ozone (O_3_) is a health damaging air pollutant [47,48,49]. The chemical formation of O_3_ in the troposphere is temperature-dependent, and higher average surface temperatures are generally expected to worsen O_3_ pollution at mid-latitudes worldwide [50,51]. The burning of solid biomass fuels to meet basic energy needs also contributes to high levels of both indoor and outdoor air pollution and exacerbates climate change [5,10,25,32,52,53].

The increasing frequency of extreme heat events driven by climate change [54,55] could also affect future energy demand [56] and air pollution levels [57]. Increasingly, the use of air conditioning is both a method to relieve exposure to oppressive heat and a status symbol of economic advancement in India [58]. Researchers estimate that sales of air conditioners are growing 20% annually and that future electricity demands for cooling could exacerbate polluting emissions from coal-fired power plants [59]. Given the current toll of air pollution and the threat of this burden increasing in the future, forward-thinking planning is vital for safeguarding public health. In particular, improved monitoring and forecasting of air quality in Indian cities and communication of this information to the public can help motivate policies to achieve a cleaner energy future, improved air quality, and a reduction in rates of air pollution-related disease and premature death [25].

### 1.3. Ahmedabad’s Leadership on Extreme Heat Preparedness

Efforts to better monitor regional air quality and communicate information about pollution and health risks in Ahmedabad build on the foundation established by the Ahmedabad Heat and Climate Study Group, which established an evidence-based Ahmedabad Heat Action Plan (HAP) and extreme heat early warning system for the city [60]. This plan, developed in response to a 2010 heat wave linked to hundreds of deaths [54], has become a template for thirty other Indian cities and eleven states working to mitigate the health risks of extreme temperatures for vulnerable populations [61]. The HAP, supported by temperature forecasts from the India Meteorological Department (IMD), helps to coordinate government agencies and public outreach activities to reduce the health risks posed by extreme heat. In 2016, the group commenced discussions to establish a parallel monitoring and risk communication system for air pollution in the city. This manuscript describes efforts related to the air pollution work; for more background information on the establishment of the HAP, see [60].

### 1.4. Objectives

The overall goal of this project was to develop a health-based strategy for monitoring and communicating information about urban air pollution in the region and the new AQI, in the form of the Air Information and Response (AIR) Plan. Specifically, the goals of this project were to: (1) Assess the current state of the regional air pollution and health evidence base and the need for an AQI; (2) improve public awareness of the air pollution problem as it relates to health; (3) identify and protect especially vulnerable groups from the health threats posed by air pollution; (4) build capacity in the medical and public health sectors for promoting health-protective strategies on air pollution; and (5) identify the future mitigation and exposure control and reduction measures with key partners from leading local institutes. To help achieve these objectives, experts were consulted in New Delhi and other cities in India and internationally to engage in knowledge exchange and information sharing on air quality monitoring policies, epidemiologic methods, and communication strategies.

The goals of this paper are to provide information on the first comprehensive air quality health risk communication and exposure mitigation plan in an Indian city, describe the challenges encountered, and highlight project aspects that may be adapted to other settings. The approach is outlined in the Methods section and project accomplishments to date are described in the Results section. The particular challenges of this work are analyzed in the Discussion section, as are the implications for other settings seeking to address the major threats to health posed by ambient air pollution.

## 2. Methods

### 2.1. Planning and Conceptual Model Development

The Ahmedabad AIR Plan builds on the effective heat action plan that the city and partnering institutions, led by the Indian Institute of Public Health-Gandhinagar (IIPH-G) and the Natural Resources Defense Council (NRDC), developed and implemented beginning in 2013 [60]. Both IIPH-G and NRDC are knowledge partners for the air and heat programs, with two separate memoranda of understanding with the city.

The conceptual model was developed based on domestic and international expert input and research documented in a comprehensive technical issue brief, which established a scientific evidence base for the eventual development of the AIR Plan [62,63,64]. Based on the background literature review, roundtable discussions, and a participatory workshop documented in the issue brief, the conceptual model is premised on several factors: Air pollution poses a significant public health risk [50]; this risk is under-recognized by the public; air pollution is inadequately monitored and managed [65]; and documenting air quality through an AQI and communicating specific exposure mitigation strategies can improve public health and facilitate long-term improvement of air quality [25,51,66]. Another underlying premise is that certain populations are at greater risk to air pollution-related health effects because of relatively high exposures (e.g., occupational exposures among outdoor workers, including traffic police, construction workers, and street vendors, among others [42,67,68]) or low coping capacity (e.g., school children, patients with pre-existing respiratory illnesses [69,70,71]). The model also posits that strategies for conveying risk information and corresponding exposure mitigation recommendations would reduce the population health risks related to air pollution [66].

### 2.2. Community Needs Assessment

The need for a comprehensive air quality and health risk communication program in the region was assessed through several measures. To gather opinions from city residents, a series of community meetings, workshops and roundtables with local stakeholders (including physicians, environmental professionals, health scientists, city staff, and other community leaders) were conducted from August 2016 to May 2017. Roundtable sessions and workshop interviews were conducted with national and international air pollution experts, local municipal administrators, and key academic institutions [62,63,72,73]. Soliciting input from members of the public as key stakeholders was critical to establish effective communication channels, mutual trust, and the reciprocal exchange of information. This approach was also conducive to forming innovative solutions, in contrast to one-way communication models [74].

### 2.3. Baseline Data

To inform efforts on improved air quality monitoring and communication, it was necessary to ascertain the information already available from prior work in the city. The peer-reviewed literature and publicly-available government reports were consulted to gauge the degree to which quantitative air quality information was available, and the epidemiological literature was surveyed for research conducted in the Ahmedabad region and the state of Gujarat.

This baseline data research involved a literature review into four aspects of air pollution data and policy: (1) India’s National Ambient Air Quality Standards (NAAQS) and the authority of the government to monitor air pollution and regulate polluting sources; (2) the extent of existing air quality monitoring systems in Ahmedabad; (3) prior research on key pollution sources and health impacts of pollution in the region; and (4) information on the operation and efficacy of other AQI systems, both in India and in other countries, with a focus on key international cities (Beijing, Los Angeles, and Mexico City).

#### 2.3.1. India’s Air Quality Standards

All AQI reporting systems relate air quality monitoring data to anticipated public health impacts by assessing monitored levels of pollution relative to health-based air quality benchmarks [23,75]. To assess the appropriate benchmarks for different AQI levels and associated public health warnings, information about India’s NAAQS was reviewed [51,76] along with documentation about the various government jurisdictions involved with monitoring, reporting, and managing air quality [25,77,78,79,80,81,82].

#### 2.3.2. Ahmedabad’s Air Pollution Monitoring

Central to efforts to gauge progress towards meeting established NAAQS are air quality monitoring networks, which characterize local conditions for multiple priority pollutants. Information was compiled about existing air quality monitoring stations in Ahmedabad, including organizational management, monitor type, parameters monitored, and the availability of monitoring data. Additionally, prior preliminary emissions inventory research in the city had begun to link polluted air to emissions from distinct sources in the city, both stationary and mobile [83]. This past research was analyzed to inform a comprehensive city-wide emissions inventory that was undertaken in 2016–2017 (See Section 3.4.5).

#### 2.3.3. Health Impacts of Air Pollution in Ahmedabad

The peer-reviewed public health literature was surveyed to better understand the burden of air pollution in Ahmedabad, as well as the need for improved air quality monitoring data at higher spatial and temporal resolution. The focus was on the quantification of health effects in vulnerable sub-populations in the city, including children [69,84], the elderly [85], people with pre-existing medical conditions [70,86,87,88] and those exposed to especially high levels of pollution due to their socioeconomic status [89,90] or occupation [67,68,91].

#### 2.3.4. Best Practices from Other AQI Systems

Best practices and fundamental elements for establishing an effective AQI system were also identified. To distill information on the key elements of effective AQI systems worldwide, the peer-reviewed academic literature, documentation from air quality management agencies, and online media sources were surveyed [22,23,24,51,66,74,92,93,94]. In particular, several dimensions of AQI systems were reviewed: Stakeholder coordination, system planning and implementation processes, communication aims and modes, system role in local capacity building, and the application of air quality monitoring data for further research.

### 2.4. Coalition Building and Outreach

Recognizing the scale and complexity of the air quality challenge in the region, a diverse array of project partners were consulted in order to broaden the team’s expertise and assist in project planning and implementation. These partners included experts from prior work on the HAP, as well as additional domestic and international stakeholders with valuable experience in air quality monitoring and health impact assessment. At the center of the coalition was the municipal city government, the Ahmedabad Municipal Corporation (AMC), which is responsible for the city’s civic infrastructure and administration. Led by the mayor and municipal commissioner, the AMC Health Department has pioneered community health strategies for decades. As an initial step for this project working with the knowledge partners, the AMC appointed a Nodal Officer within the city’s Health Department. The Nodal Officer serves as the AMC point of contact and is responsible for administering the program, including conducting robust coalition outreach along with the primary knowledge partners, IIPH-G and NRDC [60].

The Indian Institute of Tropical Meteorology’s System for Air Quality and Weather Forecasting and Research (IITM-SAFAR, [95]) program was a critical partner with deep technical expertise. IITM-SAFAR, in partnership with the AMC, helped develop and secure national funding from the Ministry of Science and Technology for the air quality monitoring systems, display boards, and communications systems. IITM-SAFAR also operates comprehensive monitoring and AQI systems in three other Indian cities (Pune, Mumbai, and New Delhi [96]) and brought its expertise and lessons learned from this work to the project in Ahmedabad.

The state-level GPCB, as part of the national Central Pollution Control Board (CPCB), was also a key asset to the coalition. The GPCB was engaged from the inception of the project, given its authority on air quality and pollution issues in the region. The GPCB also has a long-standing monitoring network in Ahmedabad and is developing a city clean air plan that aims to address priority pollution sources. The research team engaged directly with GPCB chairman and staff in developing the program, including hosting GPCB presentations and ensuring GPCB representation during coalition roundtable meetings and workshops.

Medical professionals (especially pulmonologists) from Ahmedabad and New Delhi were essential members of the coalition. Experts from AMC MET Medical College, Ashrai Associates and Sparsh Chest Diseases Center, and Apollo Hospital provided advice on the numerous risks that polluted air poses to public health with a focus on vulnerable groups [66,70,84,97]. Local physicians, pediatricians, medical officers, community-based healthcare professionals, ambulance service staff, medical colleges, urban planners, and other local environmental professionals were also engaged in the coalition and plan development process. Based on the epidemiologic research and the experiences of medical professionals, it was clear that children were especially vulnerable to air pollution [69,98,99]. As such, city educators and students were consulted to inform interventions to meet local needs [100,101]. In addition, the media played an important role in developing the AIR Plan and provided advice on effective communication and messaging to the larger community, including to disproportionately impacted groups [74,94,102].

## 3. Results

### 3.1. Planning

Effective stakeholder outreach and community engagement were essential for planning the intervention in Ahmedabad. The research team convened several planning sessions to clarify the project goals, scope, and plans for implementation. The main aim was to support the AMC and IITM-SAFAR with the new monitoring and AQI system. To inform these goals, several informal meetings and a two-day workshop on “Air Pollution & Health: Laying the Foundation for Effective Use of Ahmedabad’s Air Quality Index” were held in 2016 [62,63,72]. The workshop included local groups as well as national and international experts, as discussed in Section 2.4. After this workshop, an issue brief entitled “Protecting Health from Increasing Air Pollution in Ahmedabad” was published, which documented air quality conditions in Ahmedabad and highlighted international best practices on AQI system coordination and health risk communication [64]. The issue brief, coupled with information from the community needs assessment and background research described below, served as the scientific evidence base for eventual development of the AIR Plan.

### 3.2. Community Needs Assessment

The need for a health-based AQI and accompanying risk communication strategy was supported by several findings. Members of the public, local government leaders, academics, and other stakeholders identified a specific need during meetings, workshops, and roundtable sessions for the city to address the problem of ambient air pollution, with a focus on mitigating exposures that cause significant harm to public health [62]. Participants in these sessions also identified pollution sources of particular concern in Ahmedabad, including air pollution stemming from fires burning at a local solid waste dump [103,104], dusty roads and construction activities, the loss of vegetated areas, and the transportation sector generally [64]. Pulmonologists, pediatricians, and medical officers highlighted the need for improved environmental monitoring, forecasting, and risk communication, as did local media experts. Overall, the needs assessment made clear that community members endorsed a multi-staged air quality intervention in the city. This effort would begin with attempts to improve quantitative understanding of the problem through dramatically expanded local air quality monitoring and forecasting.

### 3.3. Baseline Data

The review of the existing literature encompassed the current state of national air policy, air pollution monitoring, health research, and AQI systems in India and other countries. This information served as the foundation for stakeholder outreach, intervention planning, and AIR Plan implementation.

#### 3.3.1. India’s Air Quality Standards

As a result of the Air Act of 1981, under the MOEFCC, the CPCB and State Pollution Control Boards (SPCBs) are responsible for establishing NAAQS and monitoring systems [76]. The Air Act mandates the CPCB and SPCBs to establish NAAQS for key criteria pollutants (PM_10_, PM_2.5_, O_3_, sulfur dioxide (SO_2_), and nitrogen oxide, (NO_2_)) and undertake nationwide air monitoring to ascertain national ambient air quality and subsequently identify the nonattainment areas where ambient pollution levels exceed the NAAQS.

CPCB adopted India’s first NAAQS in 1982 and revised them in 1994 and 2009 [95,105]. Through this system, air pollution levels are allowed to exceed the NAAQS in certain industrial, residential, rural, and other specified areas (but not in ecologically sensitive areas). SPCBs can set more stringent standards than the NAAQS for their respective states. Compared to the World Health Organization (WHO) ambient air quality guidelines and NAAQS in other countries, India’s standards are generally less stringent [106]. For example, the WHO air quality guideline for 24-h mean PM_2.5_ is 25 μg/m^3^, while the corresponding Indian standard is 60 μg/m^3^ [25,107].

In 2010, the MOEFCC issued a memorandum that declared Ahmedabad as a critically polluted area [77] and the city has periodically exceeded India’s NAAQS for PM_10_ [81]. For example, PM_10_ levels in Ahmedabad exceeded permissible limits by 30–50% for all five years between 2008 and 2012 [108,109]. In 2014, Ahmedabad was among the five most polluted cities in India for PM_2.5_ pollution and ranked in the top 15 globally [110].

#### 3.3.2. Ahmedabad’s Air Pollution Monitoring

Despite the establishment of India’s NAAQS, the country’s air is some of the most polluted in the world, according to evidence from 2015–16 [1,2,7]. Although the CPCB administers the NAAQS, national systems for documenting and reporting air quality data need to be strengthened to adequately protect the public from the health risks posed by air pollution [6,10,111,112,113]. Access to limited real-time and forecast air quality information is not available in most cities, and most directly-monitored air quality data is difficult to obtain or interpret, which stifles efforts to protect residents and inhibits research that could better quantify the health effects of harmful exposures [25,95].

As of 2016, several entities managed over twenty monitoring stations in Ahmedabad [64]. These stations were operated by several state and local organizations: nine by the GPCB (seven as part of the National Air Monitoring Programme and two for the corresponding State Programme), 14 by the Gujarat Environmental Management Institute, and one by an industrial operator, Torrent Power [27,64,114]. Three additional stations were maintained in Gujarat Industrial Development Corridor areas for the monitoring of volatile organic compounds (VOCs) only.

As part of the National Air Monitoring Programme, the GPCB manually collects ambient air pollution data from all stations in its urban network twice each week and calculates air pollution concentration averages from that data [27,113,115,116]. Specifically, over two 24-h periods each week, gaseous pollutants are sampled from continuous analyzers every four hours (SO_2_ by improved West and Gaeke method, NO_2_ by modified Jacob and Hochheiser method, and O_3_ by chemical method) [117]. Gravimetric PM is measured every eight hours using a sampler that draws ambient air at a constant volumetric flow rate (16.7 lpm) onto 47 mm Teflon filters [81]. Ambient air quality monitoring and meteorological data are compiled by both GPCB and CPCB in annual reports, available online [78,81,82,118]. Separately, CPCB maintains a single continuous urban ambient air quality monitoring station in Ahmedabad [113,119] and reports this data online in real-time [120]. Periodically, the monitoring results from the existing air monitoring stations have been unavailable online because of technical problems.

Ahmedabad’s inland location, as well as its dry and hot climate, can worsen air pollution in the city. A 2012 study evaluated air quality sources in Ahmedabad and five other Indian cities with a focus on PM_10_ [83]. The study found that the major sources for PM_10_ in Ahmedabad were road dust (30%), power plants (25%), vehicle exhaust (20%), and industry (15%), with the remainder attributed to domestic cooking and heating, diesel generators, waste burning, and construction activities. Ahmedabad’s electricity grid is supplied by two thermal coal-fired power stations. The first, the Sabarmati 400 MW power station, is situated on the Sabarmati River. The second, larger 800 MW power station is in neighboring Gandhinagar. The Sabarmati plant is one of the oldest power stations in India, operating since 1934; a 2013 study found elevated concentrations of PM, SO_2_, NO_X_, and mercury surrounding the Gandhinagar plant [121]. This research also highlighted the need for an updated and systematic city-wide emissions inventory in Ahmedabad to inform improved air pollution modeling and future policy measures aimed at emissions reductions (See Section 3.4.5).

Overall, the review of Ahmedabad’s prior air monitoring infrastructure and emissions information identified considerable gaps in the city’s understanding of local conditions and opportunities to improve its communication of health-relevant air quality information to the public.

#### 3.3.3. Health Impacts of Air Pollution in Ahmedabad

Despite Ahmedabad’s high pollution levels, the survey of peer-reviewed literature resulted in few studies specific to the air pollution-related health burden in the city. For example, in 2010, the city was estimated to experience over 4900 premature deaths attributed to excessive ambient air pollution [83]. Moreover, communities living near thermal plants are known to experience higher rates of chronic respiratory illness, asthma, cancer and premature death [122]. Monitoring of air pollution for application in epidemiologic research is limited in India, where measurements of PM_10_ are more common than for PM_2.5_, presenting challenges for accurate exposure assessment of the most dangerous fine particles [107].

Air pollution sources and resulting health risks in developing Asian cities can differ significantly from those in developed countries, in part due to the use of diesel fuel and high-sulfur content coal, as well as the widespread practice of biomass burning [123,124]. Because of this unique air quality mixture, the applicability of large-scale cohort studies that quantify the air pollution exposure-health response association in more developed countries (e.g., [11,125]) may be limited. To address India’s air pollution crisis, refinement of local epidemiologic evidence is needed. The research base in this area is steadily expanding [7,10,25,70,88,98,111,126,127,128,129,130,131,132,133,134] and future investigations would benefit from robust and transparent systems for pollution data reporting, archival, and access.

The dearth of long-term air pollution epidemiologic cohort research in India is troubling given the state of the nation’s air, the underlying population health burden, and some potentially unique genetic risks to its population. Indians have higher rates of acute myocardial infarction compared with residents of other Asian countries, but the reasons for this elevated risk are unclear [135]. This disparity holds true even for individuals at a healthy weight, suggesting that the regional genetic profile may be an independent risk factor for heart disease. Moreover, a prospective cohort study documented 31% lower levels of lung function in South Asians as compared to residents of North America and Europe [136]. Such evidence, combined with results dismissing the role of genetic risk factors in conferring risk of poor lung function, indicates the importance of better understanding environmental health determinants in India [137].

#### 3.3.4. Best Practices from Other AQI Systems

The review of other AQI systems operating in India and in other countries highlighted best practices to adapt to Ahmedabad. AQI programs started in the 1970s and now operate in over 100 cities across Asia, Australia, Europe, North America, and South America. In 2013, IITM and the IMD developed SAFAR, which provides location-specific AQI in near real-time and forecasts the daily AQI up to two days in advance. IITM-SAFAR was conceived as a major national initiative for greater metropolitan cities in India to provide local information on air quality, in collaboration with the National Centre for Medium Range Weather Forecasting [95]. IITM-SAFAR monitors are deployed in accordance with Central Pollution Control Board and World Meteorological Organization standards [95,117] and continuously collect data at 5-min intervals. IITM-SAFAR has also adopted the U.S. Environmental Protection Agency (U.S. EPA) Standard Operating Procedures for instrument calibration and maintenance [95]. IITM-SAFAR deploys state-of-the-art monitoring instruments, manufactured in France by Environnement SA [138], that are ISO standard compliant [139] and certified by U.S. EPA for their PM_10_ and PM_2.5_ sampling technology.

The IITM-SAFAR monitors represent the first network in India that continuously monitors and forecasts air pollution levels [95,140]. Monitors sample air at a height of three meters from the ground and characterize air quality for the entire city by incorporating information from sites in industrial corridors, residential areas, urban centers, agricultural zones, and areas that represent background level concentrations [95]. The monitors measure small particles (PM_2.5_ and PM_10_), O_3_, NO_2_, and carbon monoxide. The stations also monitor key meteorological parameters (ultraviolet radiation, rainfall, temperature, humidity, wind speed and direction). IITM-SAFAR publishes an AQI based on its raw monitoring data that largely corresponds to the CPCB AQI calculation methodology [51,95]. Air monitoring also informs a comprehensive, computationally intense modeling apparatus that is used to develop dynamic air quality forecasts for one and two days in advance. Section 3.4.1 further explains how IITM-SAFAR forecasts are used by the AMC Nodal Officer.

CPCB established a national AQI system in 2015 in 14 cities, and that index now summarizes air quality in 57 cities [27,112]. In addition to AQI systems administered by the CPCB and IITM, other countries have implemented their own systems to protect their citizens working in the country. For example, the U.S. embassies and consulates in New Delhi, Mumbai, Kolkata, Hyderabad, and Chennai have deployed the U.S. EPA-certified instruments and calculation methods to convert raw PM_2.5_ readings into an AQI value that helps to inform the public about air quality-related health risks [141,142]. These AQI data and health effects advisories are continuously updated online [143].

Three other cities (Beijing, Los Angeles, and Mexico City) offer experiences with AQI systems that are instructive in protecting public health and improving air quality. Beijing and many cities in China are plagued by poor air quality. To protect public health, Beijing’s AQI has been a central means for notifying communities and driving action [144]. The city’s AQI system delivers information to the public over radio, print media, hourly online reports, and a smartphone application. In tandem with better quantification of air quality, China and Beijing have instituted measures to reduce air pollution such as decommissioning and retrofitting coal-fired boilers and banning dirty cars from the road [145,146]. In California, the Los Angeles AQI is a key tool for the city’s air quality program [147]. The system facilitates health alerts on poor air quality days, broad public outreach (via television, radio, and print media), medical professional engagement, and community-based programs to raise awareness. For example, local schools operate a school flag program announcing the AQI via color-coded flags on a daily basis. In 1992, WHO identified Mexico City as the most polluted city in the world [148]. Since then, the city has made tremendous progress in understanding the polluting sources and geographic characteristics that led to particularly high air pollution levels. Mexico City’s AQI warns at-risk populations about future acute air pollution episodes and associated health risks, and was designed to generate reliable data that advances local clean air strategies [149].

In sum, effective AQI systems require strong foundations of robust air pollution monitoring, effective communication of health risk information, and robust interagency coordination. The dual goals of protecting public health from air pollution in the near-term and improving air quality over a longer time horizon are strengthened by effective AQI systems, which provide the evidence base for municipal, state, and national agencies to take policy actions to reduce air pollution.

### 3.4. Intervention Implementation

The review of the existing air quality policy landscape, available air quality data, and AQI systems operating in India and other cities worldwide informed the development the AIR Plan. Synthesizing the issue brief research and stakeholder discussions, the mayor of Ahmedabad released a draft AIR Plan at a stakeholder workshop in February 2016 [72], making it available for a three-month public comment period [73]. With the national government and coalition partners, the AIR Plan and AQI were launched jointly in May 2016 by the MOEFCC Minister [150,151]. This plan serves as the first effort in Ahmedabad to comprehensively monitor air quality and communicate information about air pollution to the public to mitigate exposures and ultimately protect health.

The plan describes a health-based governance framework designed to increase awareness, reduce exposure risk, and motivate longer-term policy action to reduce air pollution. With the IITM-SAFAR AQI as the focal point, the plan aims to facilitate information sharing on air quality, increase population preparedness for acute air pollution episodes, and improve response coordination to reduce the health impacts of air pollution on vulnerable populations. The plan’s five strategic elements are listed below further described in Section 3.4.1, Section 3.4.2, Section 3.4.3, Section 3.4.4, Section 3.4.5:

1. **Pilot Health-Based AQI Warning and Interagency Coordination**—robust interagency coordination to pilot a color-coded AQI alert system that makes air quality data from new IITM-SAFAR air quality stations in Ahmedabad available to the public.
2. **Enhanced Public Awareness and Communication Outreach**—an expansive program that communicates the AQI and protection strategies to local communities through a range of tools, including 12 new LED light board displays, hoardings and billboards; the IITM-SAFAR web portal; cellular phone text messages (SMS) and smartphone mobile application; traditional media engagement; and information, education and communication (IEC) materials translated into the local language (Gujarati).
3. **Focused Activities on Children’s Health**—development of a school flag program with local elementary schools to display colored flags corresponding to daily forecast AQI levels [101,152].
4. **Targeted Capacity Building for Medical Professionals**—engagement with private and public medical professionals to build awareness of the AQI and promote protection strategies on air pollution [66].
5. **Supporting Research on Future Exposure Reduction and Mitigation Pathways**—application of the AQI for identification of exposure mitigation and pollution source reduction measures by key academic partners from leading local institutes [152].

As a result of stakeholder outreach on air pollution in Ahmedabad, a local expert working group from regional academic and research institutes was organized to develop and recommend pollution source mitigation pathways to the AMC, GPCB, and residents in the region. This group is comprised of local leaders in government, academia, and nonprofit sectors and represents the interdisciplinary expertise that will be key to achieving sustainable actions on air pollution mitigation [152]. Section 3.4.5 further details the work of this group.

#### 3.4.1. Piloting a Health-Based AQI through Streamlined Interagency Coordination

**Figure 1:**
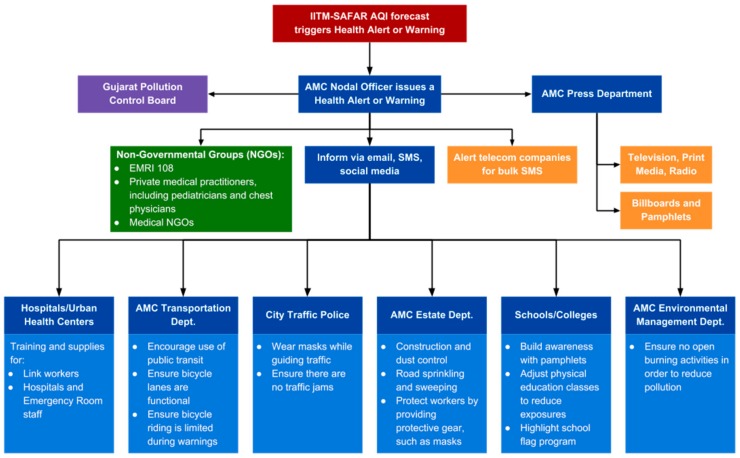
Flowchart demonstrating the array of stakeholders necessary to disseminate and act on an AQI advisory in Ahmedabad.

To develop an inclusive communication framework, the agencies already involved in responding to air-related health emergencies were visually mapped. Figure 1 shows the array of stakeholders involved in efforts to document and communicate the AQI. As the lead (Nodal) agency, the AMC Health Department has the overarching responsibility for coordination and outreach on activities related to the AQI and AIR Plan. This includes monitoring the daily AQI and disseminating public health messages to local departments and community service providers. The Health Department works with the AMC press office to promote media coverage, including in Gujarati and on social media channels, on the health risks of exposure to air pollution.

Because air pollution sources can affect large geographic areas and encompass different sectors, city and statewide coordination of government agencies is an important mechanism to effectively combat air pollution [153]. AIR Plan efforts have supported positive interagency working relationships, cooperation, and consultation. The coordination process has also created a functional framework for fostering better working relationships, improving general understanding of the work of each agency, and identifying key roles and responsibilities of each agency early in the process. Overall, coordination efforts have improved trust and understanding among agencies.

With the city of Ahmedabad, IITM activated the IITM-SAFAR network that includes 10 air pollution monitoring stations in and around the city each monitoring local conditions. Each IITM-SAFAR monitor reports pollutant-specific AQI levels based on the benchmarks specified in Table 1 for the present day (based on continuously monitored conditions), and a pollutant-specific AQI forecast for one and two days into the future. On days when levels of multiple pollutants are measured to be high, the site-level AQI is designated as the highest AQI for any specific pollutant at that site.

**Figure 2:**
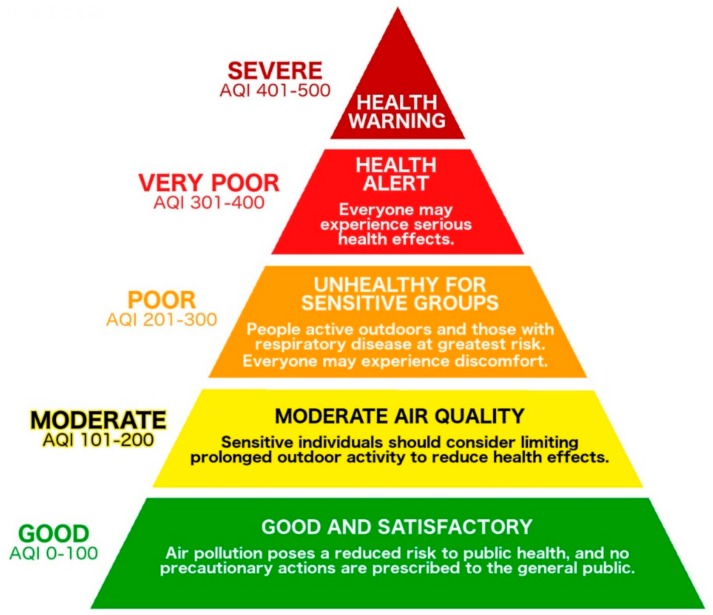
IITM-SAFAR AQI air quality descriptors, index value ranges, and associated health messages [64]. AQI levels 301–400 activate a Health Alert in Ahmedabad, while levels 401–500 activate a city-wide Health Warning.

IITM-SAFAR also calculates a city-wide average AQI based on each of the site-level AQI values, upon which Health Warnings and Alerts are based. Figure 2 displays the IITM-SAFAR air quality descriptors, AQI ranges, and associated health messages for the city-wide AQI. As part of the AIR Plan, the AMC Nodal Officer issues a Health Alert when the next day’s city-wide AQI is forecast to be Very Poor (red, AQI levels 301–400) and a Health Warning when the city-wide AQI is forecast to be Severe (maroon, AQI levels 401–500). The AMC also issues a Health Advisory bulk mobile phone text message (SMS) communication when the city-wide forecast is Poor (orange, AQI levels 201–300). Each AQI category is associated with a health advisory message, and as air quality deteriorates from the Good level, health advisory messages provide specific information about risks to public health. For example, an AQI level of 201–300 is associated with a Poor level of air quality, and indicates that sensitive populations (such as children, adults who are active outdoors, and people with respiratory disease) will experience unhealthy conditions outside. An AQI level of 301–400 (Very Poor) or 401–500 (Severe) represents a public health risk that applies to the entire population.

While the AIR Plan’s first phase focused on the dissemination of critical air quality information to the public, a longer-term effort will be required to familiarize the general population with the wide range of health effects associated with even relatively moderate levels of air pollution. Equipping the population of Ahmedabad with AQI information is the first step towards research and policy measures to better understand and eventually mitigate harmful exposures to air pollution [152].

#### 3.4.2. Conducting Effective Communication Outreach to Enhance Public Awareness

**Figure 3:**
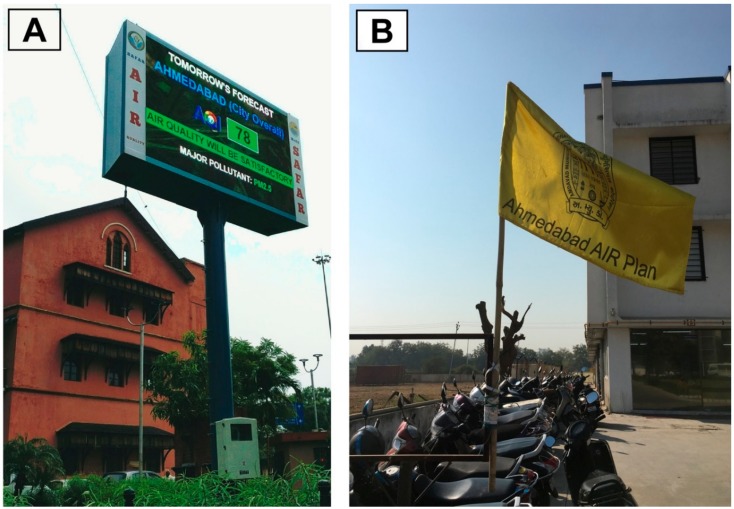
(**A**) Digital display board of IITM-SAFAR forecast city-wide AQI (one of 12 in Ahmedabad), and (**B**) A yellow flag at Zebar School for Children indicates an IITM-SAFAR AQI forecast of Moderate air quality (yellow category, AQI 101-200) for the coming day.

Essential elements for effective risk communication are information quality, transparency, simplicity, and timeliness [74,93,95,154]. In Ahmedabad, IITM-SAFAR and the AMC publicize the city-wide and site-specific AQI values through multiple channels, including online, an automated phone hotline, a mobile phone application, and a network of digital display boards that communicate the daily AQI and next-day forecast, shown in Figure 3A [140]. These display boards can help reach the considerable portion of the population for whom the internet may be unavailable, inaccessible, or unaffordable [155].

**Figure 4:**
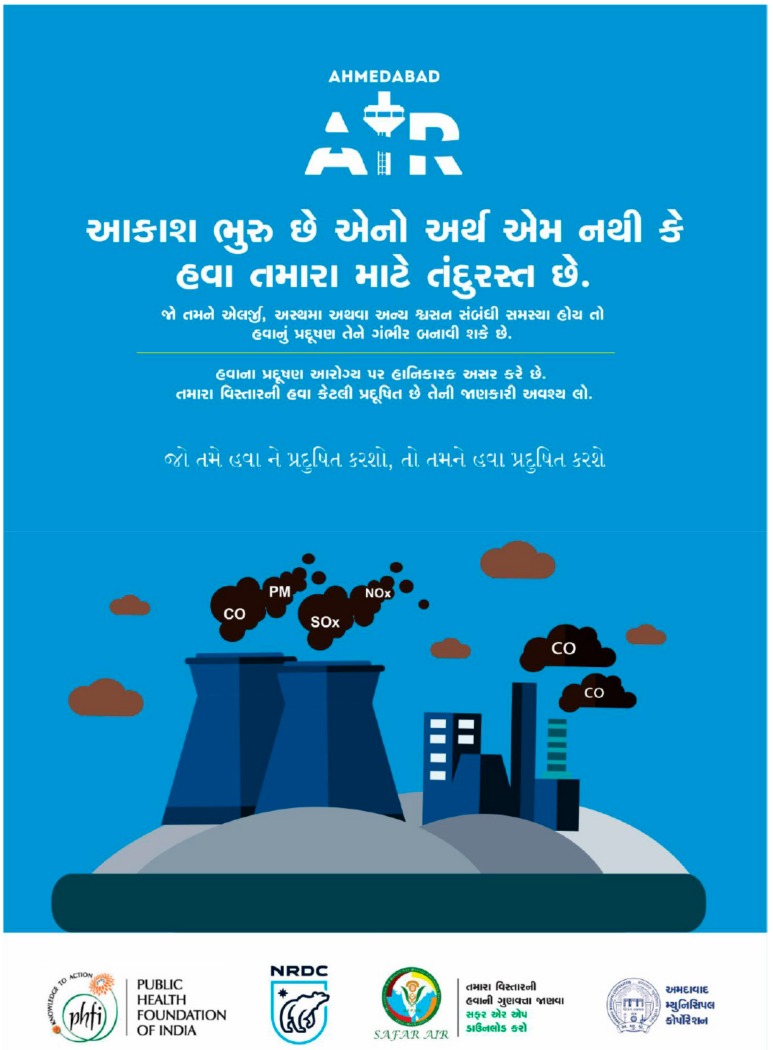
Example AIR Plan IEC outreach materials delivered in Gujarati to enhance public awareness on major air pollution sources, including thermal coal-fired power plants.

The AMC directly engages local media on the importance of air quality as a public health concern and the role of the AQI in helping to inform the public about environmental health risks. A key element of the AIR Plan’s communication strategy is the development of tailored IEC materials to explain the AQI and provide more general information about the health risks of air pollution to the residents of Ahmedabad. For example, Figure 4 shows a flyer prepared in Gujarati that helps to raise awareness about air pollution from the city’s thermal coal-fired power plants.

#### 3.4.3. Activities Focused on Vulnerable Groups

Because children are especially vulnerable to the health threats of air pollution [69,98], the AIR Plan also launched efforts to reach this population with a targeted risk communication effort [100,101,152]. Under the school flag program, the AMC provides colored flags corresponding to the five AQI categories to over 90 participating schools across the city, reaching approximately 140,000 students. A single flag is displayed at schools on weekday mornings corresponding to the city-wide AQI forecast for that day (See Figure 3B). The program also includes informative posters that educate students on the AQI pollution categories and include advice on how to modify outdoor activities when the air quality is unhealthy. The school flag program, modeled on similar initiatives in China and the United States [100], enhances student environmental literacy on the links between air quality and health and the steps that can be taken to reduce one’s exposure to pollution.

#### 3.4.4. Capacity Building Among Medical Professionals

To enhance the direct communication of the AQI through the AMC’s information dissemination channels and the media, the AIR Plan also includes strategies to deploy the expertise of health professionals, who can provide specific information on the health risks of air pollution in their consultations with patients. As trusted messengers [66], health professionals are on the front lines of advising, diagnosing, and treating respiratory and cardiovascular illnesses worsened by air pollution. These local experts can encourage especially vulnerable patients to take health-protective actions with respect to air pollution. For example, medical and health professionals can consider routinely advising patients (especially those with asthma and other respiratory or cardiovascular illness) to avoid strenuous outdoor activity when the AQI is above 200 (Moderate).

Through the AIR Plan, the AMC engages with public and private medical professionals to build health awareness and protection strategies on air pollution. Specifically, the AMC and IIPH-G are developing training sessions targeted towards medical and paramedical workforce to build capacity on air pollution and health. In addition to advocating directly for the health of individual patients, health professionals are local leaders with the experience and expertise to inform local interventions and policy strategies to reduce the health burden of air pollution.

#### 3.4.5. Initiate Research on Future Exposure Reduction and Mitigation Pathways

Ahmedabad’s work on reducing extreme heat vulnerability through its HAP has also shown that developing local scientific studies to build an evidence base on environment-health connections provides a foundation for creating and implementing effective policies [54,156]. The AIR Plan aims to support future air pollution research in the city with a focus on emissions sources, air quality trends, and epidemiologic investigation. These efforts are coordinated by the local expert working group on air pollution, which meets regularly to assess the progress of the AIR Plan and consider recommendations for longer-term air quality management in the region [157].

While some prior research has made progress towards identifying the key sources of air pollution in Ahmedabad, new monitoring efforts enabled by IITM-SAFAR allow for a better understanding of spatial variation in pollution. Upon launching the AIR Plan, the IITM-SAFAR program released its comprehensive emissions inventory assessment of the city [158]. This report showed that the top three sources of PM_2.5_ in 2016–17 were transportation (36%), industry (33%), and wind-blown dust (21%) [158]. The emissions inventory improves the precision of IITM-SAFAR’s air quality forecasts and informs ongoing policy efforts at the city level to understand and mitigate exposure to pollution from key sectors [159].

The wealth of data recorded by the IITM-SAFAR monitors allows for a more complete understanding of air quality conditions in the region. For example, spatial variation in pollution levels can be better characterized by analyzing data from the 10 monitoring stations throughout the region. Moreover, because these monitors document conditions every five minutes, stakeholders can better ascertain trends in pollution over multiple time horizons (e.g., hourly, daily, weekly, monthly, seasonally, and annually) [160,161]. As a result, the city is better equipped to understand air quality on a historical basis and potentially identify localized “hot spots” of elevated pollution levels on which to base future emission reduction and exposure mitigation actions. Such actions include concerted efforts to expand vegetated areas within the city [162], which have been shown to reduce air pollution and extreme temperatures in urban areas [163,164,165] and could also improve mental health outcomes [166,167]. Specifically, planting certain tree species can achieve substantial reductions in biogenic VOCs relative to known high-emitting species [168].

The new air quality data available in Ahmedabad can also be utilized for regional environmental health research that characterizes the risks to local citizens as a result of their exposures to pollution, particularly for PM_2.5_ [25]. As a first step, IIPH-G is planning a cross-sectional study that seeks to identify the associations between PM_2.5_ and respiratory function in children. While studies analyzing air quality and health data measured in India are underrepresented in the global air pollution epidemiology literature, improved monitoring data helps to expand the exposure evidence base for health research and environmental policy [25,70,88,98,99,111,127,128,129,130,131,132,133,134,136,169,170]. Given the unique genetic and environmental risks in the region and dose-response relationships for PM_2.5_ that are being better quantified regionally, the newly-available air pollution data collected in cities like Ahmedabad could be useful for efforts to better understand local epidemiologic risk [6,7,171,172,173]. IIPH-G also plans to apply IITM-SAFAR data in epidemiologic research examining the relationship between air quality exposure and population-level risk for hospitalization and emergency department visits.

Additional scientific studies that could serve to build the in-country evidence base include exploration of the long-term effects of air pollution exposure amongst pregnant women and newborns [174,175], the effects of fossil fuel combustion on health risks in children [97,176], and the long-term health effects and financial costs to society of chronically polluted air [3,177,178,179]. Exposure assessment research could better quantify the range of air pollution exposure among highly exposed outdoor workers (such as traffic police) and track how policy efforts to reduce emissions affect air quality exposures experienced over time [180,181]. Furthermore, air pollution data from IITM-SAFAR could improve the study of the health effects of air pollution related to heat waves in Ahmedabad, because while effects of heat on mortality have been robust to confounding by air pollution, the possibility exists for effect modification [182,183,184,185].

### 3.5. Project Evaluation

Qualitative evaluation of the AIR Plan has been conducted since its launch in 2016. Ongoing evaluation entails assessing performance of the AQI in reporting and forecasting pollution levels. It also includes assessing the effectiveness of the AIR Plan in reducing exposure of citizens to air pollution, promoting greater awareness of the health impacts of air pollution, and lowering rates of air pollution-related health problems. Over the long term, it is also important to analyze air pollution trends to understand the success of the project from a pollution mitigation standpoint [81,180].

Evaluation of the plan’s first year included assessment of two main target groups: organizations and individuals directly involved in the AIR Plan response to air pollution, and the people most vulnerable to high levels of air pollution. Impact assessment of preparedness activities in the public health community included roundtable discussions and surveys with key personnel involved in forecasting, reporting, and responding to high air pollution episodes. Specifically, understanding of AIR Plan roles and responsibilities, familiarity with preparedness and response activities, recognition of respiratory illnesses, and thoughts on barriers to implementation were evaluated. Key personnel included IITM-SAFAR experts, AMC Health Department staff, hospital administrators, urban health center staff, and administrators of emergency medical services. Table 2 describes the completed evaluation efforts corresponding to each of the AIR Plan’s five major aims.

## 4. Discussion

AMC’s launch of the AIR Plan in Ahmedabad was the culmination of a collaborative effort among an array of stakeholders. Because many Indian cities are suffering from the heavy burden of air pollution, it is important to acknowledge the local political dynamics that facilitated the planning and implementation of this plan. Locally, the AMC led the effort to make air pollution (and environmental health and resilience more generally) a municipal priority, and this proactive leadership was a major factor in the effectiveness of this work. City leaders have developed productive relationships with their counterparts in state and national government, enabling them to work collaboratively on technical and policy solutions.

Because the sources of air pollution are so numerous, a similarly diverse and interdisciplinary team was required to address the problem from multiple perspectives; The AIR Plan benefits from an engaged citizenry and enthusiastic partners and supporters across sectors. Several of the collaborating partners and institutions that worked on the AIR Plan had previously worked together to develop the HAP for the city, including the AMC, IIPH-G, IMD, and NRDC. Because of this prior work, a sense of shared accomplishment helped to propel this project forward. Moreover, the leadership of the IITM-SAFAR program allowed the research team to surmount the technical hurdles of establishing a comprehensive air quality monitoring system in the city. That IITM-SAFAR already had experience in maintaining systems in three other cities allowed its team to efficiently deploy its monitoring and AQI communication infrastructure in close coordination with the AMC.

A number of key challenges were identified during the course of background research and AIR Plan development. First, coordination of the many of stakeholders working to address air pollution has been a continuous challenge. Within the AMC, the graphical depiction of staff roles and responsibilities (See Figure 1) has been useful for clarifying tasks and avoiding duplication of effort. Beyond this visual tool, stakeholder coordination benefitted from focused and clear communications and, in the case of the expert working group, face-to-face meetings that were a more efficient medium for the exchange of ideas and information than electronic communications [152].

Second, the appropriate balancing of near, medium, and long-term objectives for actions addressing air pollution in the region presented a considerable challenge. Given time and resource constraints, the scope of the AIR Plan activities was necessarily limited. The plan was designed to enable the city to achieve tangible successes within the first year to build community support for sustained action. For example, outreach on the topic of air pollution and health focused local actions to the immediate problem of exposure reduction to protect health, while also confronting the larger challenge of mitigating polluting emissions in the first place. Likewise, local air monitoring data and the AQI were deployed in real-time public-facing communications and also regularly archived by IITM-SAFAR and IIPH-G for analysis and application in future epidemiologic research.

Third, resource challenges present a common problem to public health intervention efforts, especially because the health threats facing populations are numerous, expansive, and complex. The AIR Plan acknowledged this reality by utilizing the expertise of partners including IITM-SAFAR, IIPH-G, medical professionals, and local school leaders and empowering these groups with implementation of key aspects of the plan. In this way, project partners served as local ambassadors, reducing the administrative and financial burden on the AMC. For example, IITM-SAFAR added an Ahmedabad-specific AQI node to its online portal, email alert system, and mobile phone application in 2017. These electronic resources collectively serve as centralized repository for air quality data in the city that all stakeholders can utilize moving forward. IIPH-G helped to facilitate the execution of the school flag program and training of medical professionals, deploying its expertise in health risk communication for the benefit of project partners.

Fourth, numerous communication challenges were encountered in this work. Air pollution is a multidimensional topic, and the technical intricacies of pollution sources, ambient exposures, and health impacts can be difficult to navigate for any individual, regardless of training. Beyond the obstacles presented by these technical aspects, residents of Ahmedabad commonly speak multiple languages (dominantly Gujarati and Hindi) and approach the air pollution problem from distinct perspectives. To overcome this complexity, the team’s priority was the delivery of clear, consistent messages tailored to specific audiences. For example, IEC materials developed in support of the AIR Plan were focused on explaining the air pollution challenge, the known impacts of pollution on health, and ways for citizens to lessen their exposures to polluted air.

Looking into the future, the AIR Plan stakeholders plan to periodically reconvene the local interdisciplinary expert working group to prioritize, research, and recommend policy interventions that could target specific sources of air pollution in Ahmedabad, based on newly-available air pollution data [27,152]. These efforts could also include an analysis of monitored conditions in Ahmedabad relative to India’s NAAQS and the National Clean Air Programme. Such data could be used to strengthen NAAQS enforcement mechanisms, thereby enhancing the health benefits delivered by AQI reporting [24]. The relationship between air pollution regulations and economic development, while sometimes a point of tension, could also be analyzed in further detail with air quality data at a high spatial resolution that illuminates the major personal and societal costs associated with adverse health impacts [3,177,178,187]. Moreover, policymakers could consider specific policy measures to deploy on days with particularly high levels of pollution, such as restrictions on industrial activity or vehicular traffic [181]. Another area for policymakers to consider is the AQI threshold at which communication to vulnerable groups in the city is initiated (See Figure 2), and whether this threshold should be lowered as the AIR Plan implementation continues in order to further protect public health.

## 5. Conclusions

The experience of developing and implementing the AIR Plan in Ahmedabad offers a number of tangible lessons to other cities in India and other countries that are striving to address the problem of urban air pollution. First, strong partnerships and community engagement are paramount. Second, the reliance on transparent, tested technology for documenting and reporting air quality builds trust and allows for the establishment of a research base on which to craft informed air quality management policies. Third, it is important to design interventions that achieve near-term benefits while also laying the foundation for sustainable progress towards larger environmental and public health goals. Lastly, a sustained, stakeholder-focused approach that prioritizes community engagement at the outset is vital.

As cities around the world strive to achieve healthy and sustainable conditions for their residents, Ahmedabad’s leadership with the AIR Plan demonstrates that the combined efforts of government agencies, health professionals, academic leaders, and engaged community members can serve to effectively inform the public about major air pollution-related health risks. This project also lays a foundation for policy measures to improve the city’s resilience to other complex environmental health threats. The lessons learned through the development and implementation of Ahmedabad’s AIR Plan are instructive for other cities working to address the heavy burden of air pollution on the health of their citizens.

## Figures and Tables

**Table 1:** IITM-SAFAR air quality descriptors, AQI numeric values, and corresponding air pollution concentration thresholds and temporal averaging periods [95,140].

Air Quality Descriptor	AQI Value	PM_2.5_ (μg/m^3^) 24-hr Average	PM_10_ (μg/m^3^) 24-hr Average	O_3_ (ppb) 8-hr Average	NO_2_ (ppb) 24-hr Average	CO (ppm) 24-hr Average
Good	0–100	0–60	0–100	0–50	0–43	0–1.7
Moderate	101–200	61–90	101–250	51–84	44–96	1.8–8.7
Poor	201–300	91–120	251–350	85–104	97–149	8.8–14.8
Very Poor	301–400	121–250	351–430	105–374	150–213	14.9–29.7
Severe	401–500	251–350	431–550	375–450	214–750	29.8–40

**Table 2:** Key aims of the AIR Plan and corresponding evaluation methods.

AIR Plan Aim [151]	Evaluation Method
1. Health-Based AQI Warning and Interagency Coordination	Meetings with AMC and IITM-SAFAR staff and development of a draft internal AIR Plan User Guide and Standard Operating Procedures to standardize and strengthen interagency coordination practices.
2. Communication and Outreach	Community roundtable meetings to qualitatively gauge the success of communication and outreach efforts. Development additional public outreach materials and engagement of local media on the AQI and AIR Plan.
3. Focused Activities for Vulnerable Groups	Roundtable discussions with school administrators participating in the school flag program to assess student understanding and engagement [152].
4. Capacity Building of Medical Professionals	Conversations with leading medical professionals [152,186].
5. Research on Exposure Reduction and Mitigation Pathways	Local expert working group discussions of IITM-SAFAR AQI data and the Emissions Inventory to inform emission reduction efforts [24,152,158].

## Abbreviations

AIR	Ahmedabad Air Information and Response Plan
AMC	Ahmedabad Municipal Corporation
AQI	Air quality index
CPCB	Central Pollution Control Board
GPCB	Gujarat Pollution Control Board
HAP	Heat Action Plan
IEC	Information, education, and communication materials
IIPH-G	Indian Institute of Public Health-Gandhinagar
IITM-SAFAR	Indian Institute of Tropical Meteorology System of Air Quality and Weather Forecasting and Research
IMD	India Meteorological Department
MOEFCC	Ministry of Environment, Forests, and Climate Change
NAAQS	National Ambient Air Quality Standards
NRDC	Natural Resources Defense Council
NO_2_	Nitrogen dioxide
O_3_	Ozone
PM	Particulate matter
PM_2.5_	Fine particulate matter, aerodynamic diameter ≤2.5 microns
PM_10_	Coarse particulate matter, aerodynamic diameter ≤10 microns
SO_2_	Sulfur dioxide
SPCB	State Pollution Control Board
U.S. EPA	U.S. Environmental Protection Agency
VOCs	Volatile organic compounds
WHO	World Health Organization
